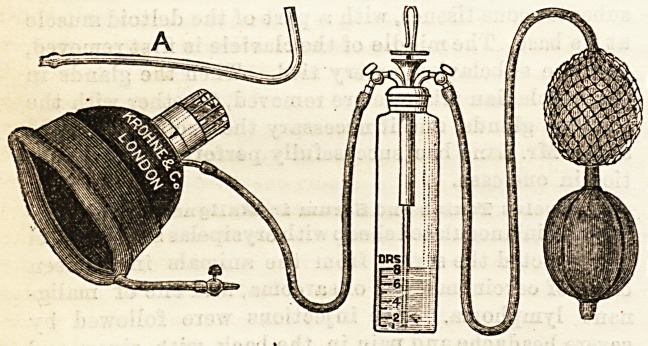# New Appliances and Things Medical

**Published:** 1895-12-21

**Authors:** 


### NEW APPLIANCES AND THINGS MEDICAL.

[We shall be glad to receive, at our Office, 428, Strand, London, W.C., from the manufacturers, specimens of all new preparations and appliances, which may be brought out from time to time.]

### IMPROVED CHLOROFORM INHALER.

(Krohne and Sesemann, 8, Duke Street, Manchester Square, W.)

This new apparatus, as may be seen from the diagram, is on the principle of Junkers' inhaler, with, however, several most important modifications. It differs from all its predecessors in that it allows of an accurate administration of the anæsthetic. The apparatus has been empyrically graduated, and from carefully repeated experiments it has been found that each full compression of the bellows evaporates at a temperature of 62 deg. Fahr. one minim of chloroform, and the amount of chloroform evaporated corresponds exactly to the degree of pressure exercised on the ball—for instance, if it is only half emptied each compression represents half a minim evaporated, and so on. By means of a feather indicator it is easy to time each compression so that it corresponds to the inspiratory phase of the patient's breathing, and in this way no choloroform is wasted and the patient takes the entire quantity into his lungs. Experience has taught that if this rule is carefully followed the following amount of chloroform is sufficient to induce anæsthesia. During the first minute of the administration at each inspiration only one-eighth of the contents of the bellows should be emptied by a corresponding pressure in the ball; during the second minute one-quarter of the contents should be emptied, during the next minute one-half, and the next minute three-quarters. By this time anæsthesia is usually induced. Some patients may require a further supply, and in such cases the bellows must be completely compressed during each inspiration until narcosis ensues. From this time onwards anæsthesia may be maintained by very small doses of vapour, sufficient in fact to replace what is lost by exhalation from the lungs. The opening of the delivery tube in the mouthpiece is directed so that the stream of chloroform vapour plays on the oral and nasal orifices, and consequently none is lost in the surrounding air. There is a free and unimpeded entry of atmospheric air, and the feather indicator offers practically no resistance to the exit of air from the lungs. In every way this new apparatus commends itself to us, and we believe that by its use the administration of chloroform will be reduced to simple formula with mathematical accuracy and that the dangers of this anæsthetic will be reduced to a minimum.

### AIR-TIGHT RECEPTACLES FOR DRESSINGS.

(Severin Immenkamp, Chemnitz. Agent: M. Sella, 15, City Road, E.C.)

It is easy enough to manufacture air-tight cases suitable for antiseptic or aseptic dressings, but the chief obstacle to their general acceptance by surgeons hitherto has been the expense, which in some cases has been considerable. Messrs. Severin Immenkamp have, however, gone far to solve this difficulty by their new method of manufacturing these air-tight receptacles. The cases are, for the most part, cylindrical in shape and made of parchment paper or similar material which is air and water tight, with sheet metal ends securely fastened by air-tight joints. The paper or parchment can easily be cut through with a knife or a pair of scissors, and hence obviates the necessity of using any form of tin-opener, an instrument which is required for the old-fashioned sheet metal receptacle. A second variety of case, also patented by this firm, consists of two halves, which can be soldered together with a special band of sheet metal, which can be removed when desired by tearing off with a special instrument. The ends are swaged on to the sides or shell of the box, and may be lined with discs of some antiseptic material, and the entire case may be lined in the same manner with some suitable packing material. It is claimed for this new patent that antiseptic or aseptic dressings can be packed in these cases without fear of accidental contamination from the air, as is very liable with the methods in general use. Its great merit, however, consists in its cheapness.

## Figures and Tables

**Figure f1:**